# CXCL12 Mediates Aberrant Costimulation of B Lymphocytes in Warts, Hypogammaglobulinemia, Infections, Myelokathexis Immunodeficiency

**DOI:** 10.3389/fimmu.2017.01068

**Published:** 2017-09-04

**Authors:** Giuliana Roselli, Elisa Martini, Vassilios Lougaris, Raffaele Badolato, Antonella Viola, Marinos Kallikourdis

**Affiliations:** ^1^Adaptive Immunity Laboratory, Humanitas Clinical and Research Center, Rozzano, Italy; ^2^Department of Pediatrics, Institute of Molecular Medicine Angelo Nocivelli, University of Brescia, Brescia, Italy; ^3^Department of Biomedical Sciences, University of Padova, Padova, Italy; ^4^Venetian Institute of Molecular Medicine, Padova, Italy; ^5^Humanitas University, Rozzano, Italy

**Keywords:** WHIM immunodeficiency, B cells, activation, chemokine, costimulation

## Abstract

The Warts, Hypogammaglobulinemia, Infections, Myelokathexis (WHIM) syndrome is an immunodeficiency caused by mutations in chemokine receptor CXCR4. WHIM patient adaptive immunity defects remain largely unexplained. We have previously shown that WHIM-mutant T cells form unstable immunological synapses, affecting T cell activation. Here, we show that, in WHIM patients and WHIM CXCR4 knock-in mice, B cells are more apoptosis prone. Intriguingly, WHIM-mutant B cells were also characterized by spontaneous activation. Searching for a mechanistic explanation for these observations, we uncovered a novel costimulatory effect of CXCL12, the CXCR4 ligand, on WHIM-mutant but not wild-type B cells. The WHIM CXCR4-mediated costimulation led to increased B-cell activation, possibly involving mTOR, albeit without concurrently promoting survival. A reduction in antigenic load during immunization in the mouse was able to circumvent the adaptive immunity defects. These results suggest that WHIM-mutant CXCR4 may lead to spontaneous aberrant B-cell activation, via CXCL12-mediated costimulation, impairing B-cell survival and thus possibly contributing to the WHIM syndrome defects in adaptive immunity.

## Introduction

The Warts, Hypogammaglobulinemia, Infections, Myelokathexis (WHIM) syndrome is a rare congenital immunodeficiency characterized by myelokathexis, hypogammaglobulinemia, recurrent sino-pulmonary infections, and human papillomavirus-induced warts. Almost all cases of WHIM syndrome have been shown to be caused by dominant mutations in the last 10–19 amino acids of the carboxy-terminal domain (C-terminus) of the chemokine receptor CXCR4 (1, 2). The mutations interfere with ligand-induced internalization of the receptor, leading to increased signaling responses to the cognate chemokine ligand of CXCR4, CXCL12 (1, 3, 4). As a result, increased neutrophil responsiveness to CXCL12 is thought to drive retention of neutrophils in the bone marrow (myelokathexis), where CXCL12 is highly expressed, leading to their scarcity in the periphery (neutropenia) (5, 6). The causes of many of the remaining WHIM symptoms, such as B cell lymphopenia, hypogammaglobulinemia, and impaired antibody somatic hypermutation and isotype switching (7–9) are indicative of adaptive immune system defects, involving T-cell and B-cell functions. These have yet to be fully explained.

Previous studies have shown that, given the broad expression of CXCL12, WHIM-associated mutations in CXCR4 cause changes in lymphoid compartment architecture, where adaptive responses are initiated, thus possibly affecting adaptive immune function (8, 10, 11). On a more functional level, we have recently demonstrated that WHIM-associated mutations in CXCR4 reduce the stability of immunological synapses, the junctional structures between T cells and antigen-presenting cells (APC) that are essential for adaptive immune response initiation. In the absence of stable synapses, WHIM T cell activation and T cell-dependent B cell functions, such as isotype switching, are impaired (12, 13).

Here, we sought to extend our analysis of WHIM-related defects to B cell function. We examined peripheral blood B cells from WHIM patients as well as from a CXCR4^+/1013^ knock-in mouse model of WHIM (10). The WHIM knock-in mice have been shown to feature B cell lymphopenia (10, 11), similar to patients. A recent report demonstrated that the B cell lymphopenia in WHIM knock-in mice originates from an immune cell-intrinsic defect (11). Despite the B cell lymphopenia, CXCR4^+/1013^ knock-in mutant B cells were shown to be more responsive to immunization, leading to higher accumulation of antigen-specific plasma cells in the spleen though not the bone marrow. Yet, the long-term antibody titers post-immunization did not show a matching increase (11). Aiming to find mechanistic evidence that could better explain these contrasting findings, we hypothesized that WHIM-mutant CXCR4 could be leading to an aberrant B cell activation, which could then contribute to lymphopenia and the subsequent immune impairment in WHIM.

In the work presented here, we identify, in WHIM patients as well as in WHIM knock-in mice, that B cells are characterized by increased propensity for apoptosis and by a spontaneous expression of activation markers, in the absence of exogenous experimental stimulation. This could be explained by a novel costimulatory effect of CXCL12 on WHIM-mutant but not wild-type (WT) B cells. CXCL12 costimulation in WHIM, possibly via mTOR, led to increased B cell activation, however without the concurrent triggering of pro-survival signals. Finally, we found that a reduction in antigenic load during immunization in WHIM knock-in mice could circumvent the defects in WHIM B cell function. These findings identify a putative novel, mechanistic explanation for the complex adaptive response defects in WHIM.

## Results

### B Cell Immunodeficiency in WHIM Patients and Knock-In Mice Is Associated with Increased Apoptosis and Spontaneous B Cell Activation

**Figure 1:**
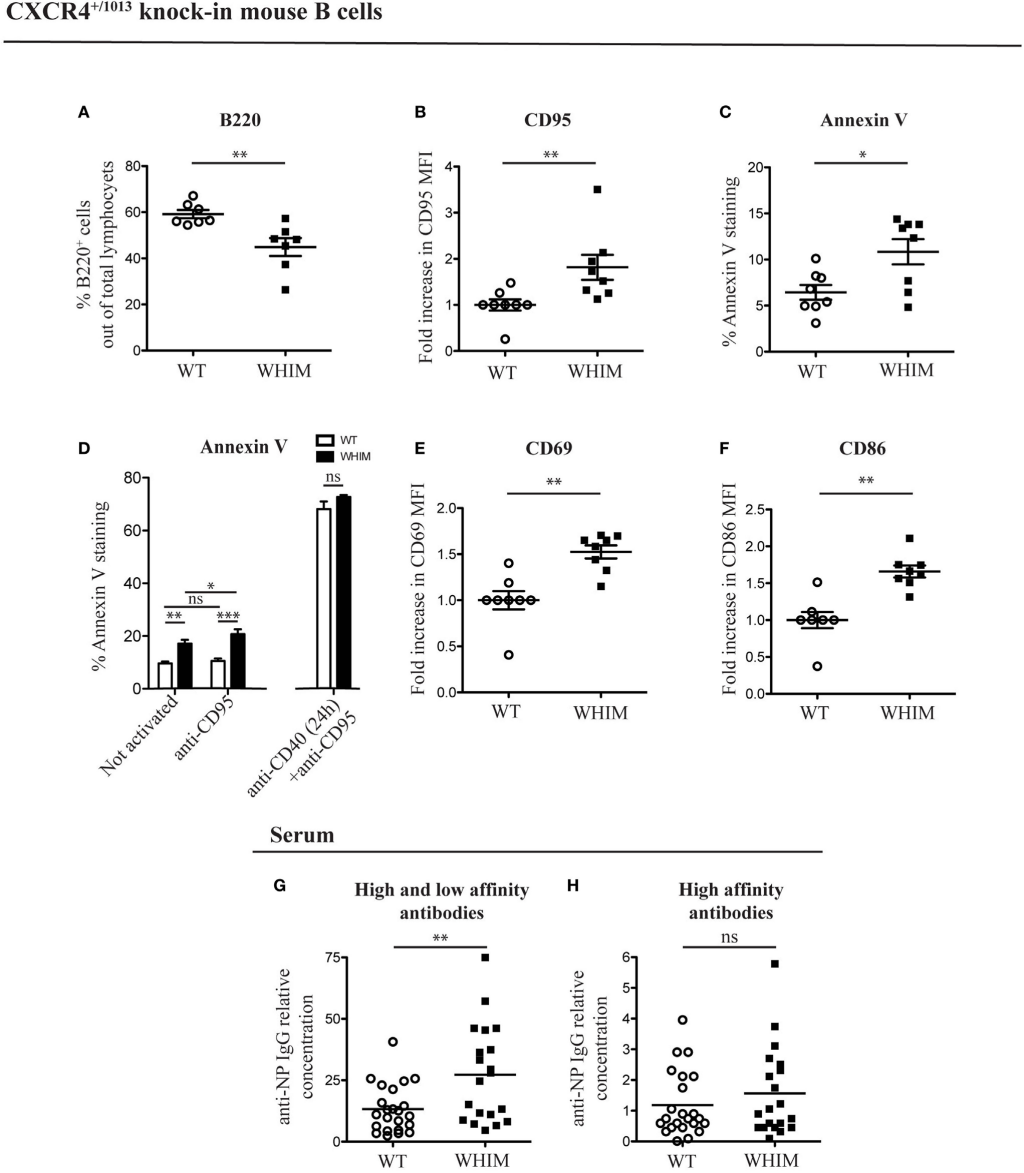

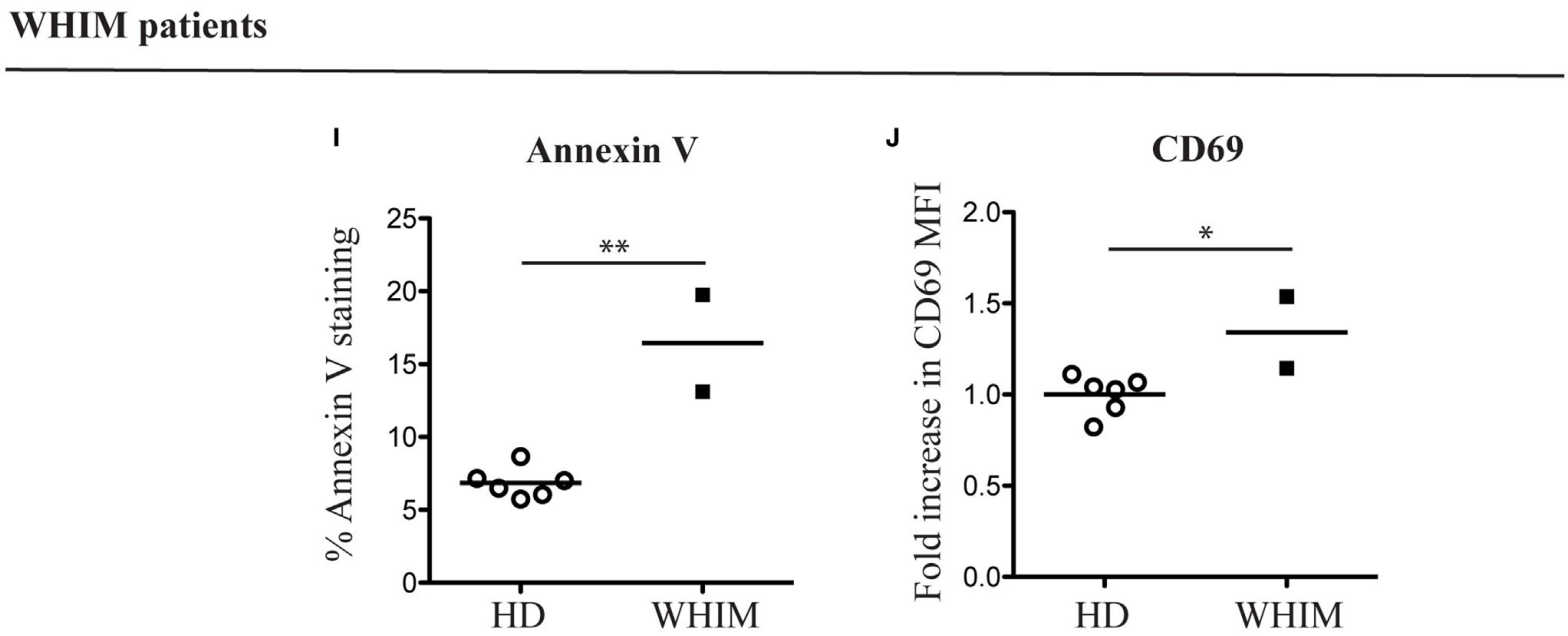
Warts, Hypogammaglobulinemia, Infections, Myelokathexis (WHIM) B cells show higher levels of apoptotic and activation markers in the absence of immunization, in mouse and human. **(A)** B-cell frequency in the spleen of wild-type (WT) and WHIM knock-in mice. Summary of *n* = 7 WT mice and 7 WHIM mice. **P < 0.01, unpaired *t*-test after positive outcome of normality testing. **(B)** CD95 staining in unactivated splenic B cells from WT and WHIM knock-in mice. Summary of *n* = 8 WT and 8 WHIM mice. **P < 0.01, Mann–Whitney non-parametric test after negative outcome of normality testing. Fold increase was used to ease comparison across independent experiments. **(C)** Annexin V staining in unactivated splenic B cells from WT and WHIM knock-in mice. Summary of *n* = 8 WT and 8 WHIM mice. *P < 0.05, unpaired *t*-test after positive outcome of normality testing. **(D)** Purified WT (white bars) or WHIM (black bars) splenic B cells were stimulated or not with anti-CD95 (250 ng/ml) for 24 h and analyzed for binding of apoptosis marker Annexin V, by FACS (left segment). Summary of *n* = 4 WT and 4 WHIM mice. As an additional control, CD95 up-regulation was induced by pre-treating B cells with anti-CD40 (1 µg/mL) for 24 h (right segment). ns: not significant; *P < 0.05, **P < 0.01, ***P < 0.001 (two-way ANOVA and Bonferroni’s post-test). **(E)** CD69 expression in unactivated splenic B cells from WT and WHIM knock-in mice. Summary of *n* = 8 WT and 8 WHIM mice. **P < 0.01, Mann–Whitney non-parametric test after negative outcome of normality testing. Fold increase was used to ease comparison across independent experiments. **(F)** CD86 expression in unactivated splenic B cells from WT and WHIM knock-in mice. Summary of *n* = 8 WT and 8 WHIM mice. **P < 0.01, Mann–Whitney non-parametric test after negative outcome of normality testing. Fold increase was used to ease comparison across independent experiments. **(G)** Relative concentration of high and low affinity antibodies (anti-NP IgG against NP_>20_–BSA) in unactivated WT and WHIM knock-in mice serum, evaluated by enzyme-linked immunosorbent assay (ELISA) (*n* = 23 WT and *n* = 20 WHIM mice). **P < 0.01, Mann–Whitney non-parametric test after negative outcome of normality testing. **(H)** Relative concentration of high-affinity antibodies (anti-NP IgG against NP_1–9_–BSA) in unactivated WT and WHIM knock-in mice serum, evaluated by ELISA (*n* = 23 WT and *n* = 20 WHIM mice). ns: not significant; Mann–Whitney non-parametric test after negative outcome of normality testing. See Section “Experimental Procedures” for details on the units shown. **(I)** Annexin V staining in CD19^+^ unactivated peripheral blood lymphocytes from healthy donors (HD) and WHIM patients. Summary of *n* = 6 HD and 2 WHIM individuals. **P < 0.01, unpaired *t*-test after positive outcome of normality testing. **(J)** CD69 expression in CD19^+^ unactivated peripheral blood lymphocytes from HD and WHIM patients. Summary of *n* = 6 HD and 2 WHIM individuals. *P < 0.05, unpaired *t*-test after positive outcome of normality testing. Fold increase was used to ease comparison across independent experiments.

In order to analyze the B cell response defects that characterize the WHIM syndrome, we utilized a knock-in mouse strain (CXCR4^+/1013^) that harbors a WHIM-associated dominant mutation of the Cxcr4 gene (WHIM knock-in mice) (10). Previous characterization of WHIM knock-in mice revealed the presence of panleukopenia, in similarity to WHIM patients. Frequency and absolute numbers of splenic WHIM-mutant B220^+^ B cells have been found to be reduced (10, 11). This reduction was attributed to a cell-intrinsic defect of WHIM-mutant lymphocytes (11). We confirmed the presence of B cell lymphopenia, via flow cytometry, by identifying reduced B cell frequencies in spleens of WHIM knock-in mice compared to WT controls (Figure 1A; absolute numbers shown in Figure S1A in Supplementary Material). To assess if the reduced splenic B cell frequency could be due to increased apoptosis, we looked for the expression of pro- and anti-apoptotic markers. B cell activation and costimulation under optimal conditions promote B cell survival through the expression of anti-apoptotic genes, such as Bcl-x (also termed Bcl-x_L_) and Bcl-2 (14). CD95 up-regulation can also occur in B cells after CD40L-mediated costimulation, rendering the B cell eventually susceptible to apoptosis if it encounters the cognate ligand, CD95L (15). In WHIM-mutant B cells, we observed significantly increased basal expression of CD95 (Figure 1B) and a down-regulation of Bcl-x (Figure S1B in Supplementary Material), showing higher susceptibility to apoptosis. Anti-apoptotic gene Bcl-2 was similarly down-regulated in WHIM B cells (Figure S1C in Supplementary Material). Examining molecules further downstream in the pathway of apoptosis, Caspase 3/7 activity, a hallmark of caspase-dependent apoptosis (16), was significantly higher in WHIM B cells compared to WT controls (Figure S1D in Supplementary Material). Finally, increased Annexin V staining in WHIM-mutant B cells identified an increased loss of cell membrane integrity (Figure 1C). We identified a similar phenotype in peripheral B cells of two WHIM patients carrying the CXCR4 (C1000T) mutation. The WHIM patient B cells showed increased Annexin V staining compared to healthy donor controls (Figure 1I). Taken together, these results demonstrate that WHIM-mutant B cells are more prone to apoptosis.

The increased expression of CD95 in WHIM B cells could suggest, as mentioned above, increased susceptibility to apoptosis upon encounter of the physiological ligand, CD95L. *In vitro* administration of CD95L led to a significant, yet limited, increase in Annexin V staining in WHIM but not WT B cells (Figure 1D, left segment). CD95L administration also increased the significance of the difference between WHIM and WT B-cell Annexin V staining. While these observations are compatible with increased baseline propensity for cell death in WHIM, it should be noted that standard CD95L triggering assays are usually performed after a 24-h pre-treatment with anti-CD40, which forces CD95 up-regulation (17, 18). When we performed such a pre-treatment, the pro-apoptotic effect was of such magnitude in both groups so as to eliminate any differences (Figure 1D, right segment).

Cell death can occur as a consequence of activation in the absence of cell survival signals, hence we examined the expression of activation markers in WHIM B cells. In the mouse model, we observed significantly higher expression of cell surface activation marker CD69 in WHIM-mutant B cells compared to WT B cells (Figure 1E). Similarly, the B-cell activation marker CD86 was also expressed at significantly higher levels (Figure 1F). Although we performed our analyses in the absence of any activating stimulus, up-regulation of both these markers suggests that the WHIM B cells have been activated *in vivo* via antigen triggering of their B cell receptor (BCR) (19).

The mice examined were housed in specific pathogen-free conditions, whereas humans are more likely to be continuously exposed to antigens. Thus, a higher activation state in human lymphocytes would be expected. Nonetheless, similarly to mouse WHIM-mutant B cells, the activation level of WHIM patient B cells was significantly higher than that of healthy donor controls, as evidenced by the increased expression of activation marker CD69 (Figure 1J).

Warts, Hypogammaglobulinemia, Infections, Myelokathexis knock-in mice have been recently shown to accumulate, after *in vivo* immunization, increased numbers of total plasma cells in the bone marrow but not the spleen, while increased numbers of antigen-specific plasma cells were found in the spleen but not the bone marrow (11). Surprisingly, in the absence of any applied activating stimulus, we found significantly higher frequency of CD138^+^ plasma cells in the spleen (Figure S1E in Supplementary Material) but not in the bone marrow (Figure S1F in Supplementary Material) in WHIM-mutant mice compared to controls. As B cells differentiate into plasma cells only after activation, our unexpected finding lends further support to the suggestion that a spontaneous activation could be occurring in WHIM-mutant B cells. To further investigate this, we examined the serum of naive WHIM knock-in or WT mice for the presence of IgG antibodies against the model hapten NP. The B-cell repertoire includes naturally occurring BCR specificities that cross-react with NP (20), though in the absence of activation and B-cell maturation, no high-affinity antibodies would be expected. By using enzyme-linked immunosorbent assay (ELISA), testing binding to high-avidity NP-coated versus low-avidity NP-coated carrier protein, we were able to differentiate between the titers of antibodies with mere ability to recognize NP versus high-affinity NP binders, respectively (21). Confirming the observed spontaneous activation in WHIM, we found significantly higher titers of anti-NP antibodies in WHIM knock-in compared to WT mice (Figure 1G); however, high-affinity anti-NP IgG serum titers were similar in the two groups (Figure 1H).

Thus, WHIM-mutant B cells appear to be spontaneously activated, expressing surface activation markers as well as producing antibodies, albeit of only low specificity. Altogether, these findings suggest that the aberrant spontaneous activation in mouse and human WHIM-mutant B cells is associated with increased propensity for apoptosis.

### CXCL12 Uncouples Signals of Activation and Survival in WHIM-Mutant B Cells

**Figure 2:**
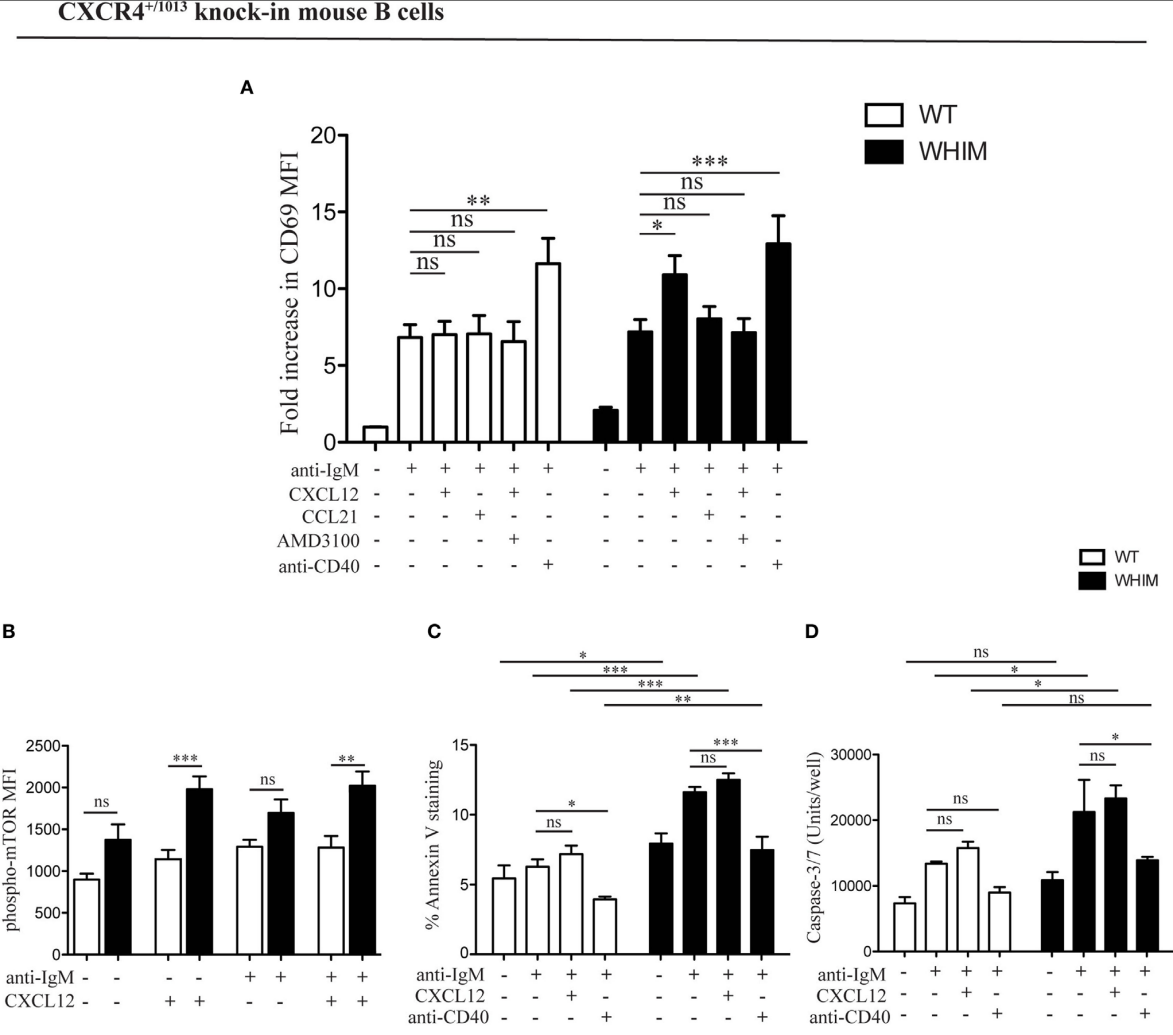

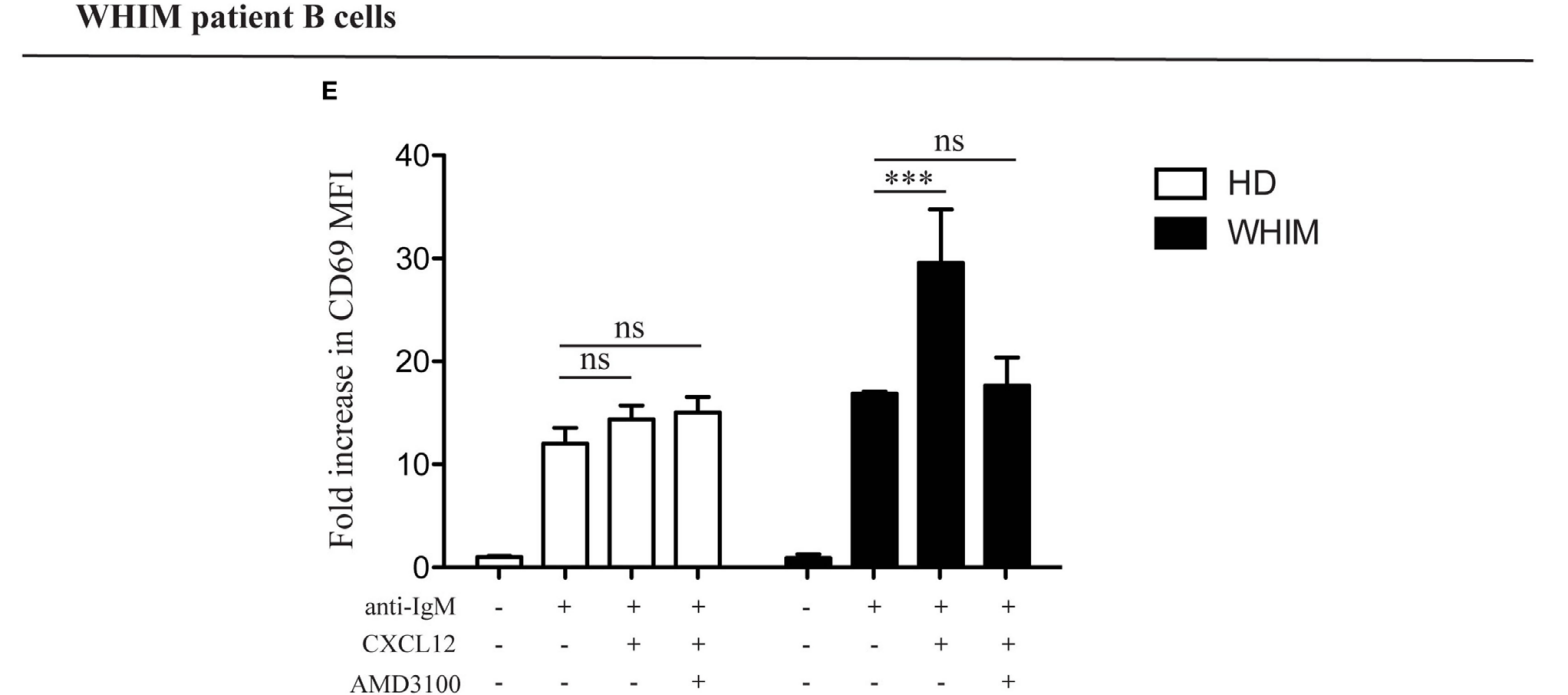
CXCL12 mediates costimulation in Warts, Hypogammaglobulinemia, Infections, Myelokathexis (WHIM) but not in wild-type (WT) B cells, via mTOR, uncoupling activation from survival signals. **(A)** Purified WT (white bars) or WHIM (black bars) splenic B cells were stimulated with anti-IgM (10 µg/ml) ± CXCL12 (25 nM) ± CCL21 (25 nM) ± AMD3100 (10 µg/mL) ± anti-CD40 (1 µg/mL). Expression of early activation marker CD69 was evaluated after 18 h by FACS. Summary of *n* = 5 WT and 5 WHIM mice. ns: not significant; *P < 0.05, **P < 0.01, ***P < 0.001 (two-way ANOVA and Bonferroni’s post-test). **(B)** Purified WT (white bars) or WHIM (black bars) splenic B cells were stimulated with anti-IgM (10 µg/ml) ± CXCL12 (25 nM). Expression of phospho-mTOR was evaluated after 5 min by FACS. Summary of *n* = 5 WT and 4 WHIM mice. ns: not significant; **P < 0.01, ***P < 0.001 (two-way ANOVA and Bonferroni’s post-test). A converse ANOVA within each group yields the following results: WT not activated vs CXCL12: * (*P* < 0.05); WHIM not activated vs CXCL12: ***; WHIM CXCL12 vs WHIM anti-IgM: *; WHIM anti-IgM vs WHIM anti-IgM CXCL12: **. **(C)** Purified WT (white bars) or WHIM (black bars) splenic B cells were stimulated with anti-IgM (10 µg/ml) ± CXCL12 (25 nM) ± anti-CD40 (1 µg/mL). Binding of marker of apoptosis Annexin V was evaluated by FACS after 18 h. Summary of *n* = 4 WT and 4 WHIM mice. ns: not significant; *P < 0.05, *P < 0.01, ***P < 0.001 (two-way ANOVA and Bonferroni’s post-test, inter-group and intra-group). **(D)** Purified WT (white bars) or WHIM (black bars) splenic B cells were stimulated with anti-IgM (10 µg/ml) ± CXCL12 (25 nM) ± anti-CD40 (1 µg/mL). Caspase 3/7 activity was evaluated by luminescence after 24 h. Summary of *n* = 3 WT and 3 WHIM mice. ns: not significant; *P < 0.05 (two-way ANOVA and Bonferroni’s post-test). **(E)** Purified healthy donor (HD) (white bars) or WHIM (black bars) peripheral blood lymphocytes were stimulated with anti-IgA/D/M (10 µg/ml) ± CXCL12 (25 nM) ± AMD3100 (10 µg/mL). Expression of early activation marker CD69 was evaluated within the CD19^+^ population after 18 h by FACS. Summary of *n* = 6 HD and 2 WHIM individuals. ns: not significant; ***P < 0.001 (two-way ANOVA and Bonferroni’s post-test).

We have previously demonstrated the capacity of CXCR4 to convey costimulatory signals to T cells (22, 23), though no such CXCL12-mediated effect has been described for B cells. Given the aberrant spontaneous activation of WHIM-mutant B cells, we thus wondered whether CXCL12 could mediate costimulation in B cells. When added to WT B cell cultures at a concentration previously shown to be functional *in vitro* (2, 12), CXCL12 was unable to provide any costimulatory effect as measured by CD69 up-regulation 18 h post-activation (Figure 2A, left panel). However, the activation of B cells derived from WHIM knock-in mice was significantly enhanced by CXCL12 (Figure 2A, right panel, representative experiment also shown in Figure S2A in Supplementary Material), demonstrating that CXCL12 can costimulate B cells expressing WHIM-mutant CXCR4. The costimulatory effect was specific for CXCL12, as the chemokine CCL21 did not have any costimulatory effect (Figure 2A, right panel). Furthermore, the effect of CXCL12 was blocked by addition of the competitive inhibitor of CXCR4, AMD3100, proving that the effect occurs via the receptor (Figure 2A, right panel). As expected, costimulation via the most-well-characterized receptor, CD40, boosted the activation of both WHIM- and WT B cells (Figure 2A). Similar results were obtained when examining the up-regulation of CD86 (Figure S2B in Supplementary Material). Notably, administration of CXCL12 in the absence of BCR-triggering did not have any effect on B-cell activation, as measured by CD69 (Figure S2C in Supplementary Material).

In order to identify the molecular pathway via which this costimulation signal could be transduced, we examined the phosphorylation levels of mTOR, a kinase in the Akt pathway, lying downstream of both BCR and CXCR4 signaling cascades (11, 24). Signals through a shared pathway could enable CXCR4-mediated signaling to boost BCR activation signals. Akt phosphorylation in WHIM B cells was recently shown to be enhanced by 5 min of CXCL12 administration in WHIM but not WT B cells, independent of BCR crosslinking (11). Confirming and extending these results, we found that mTOR phosphorylation was significantly increased 5 min after CXCL12 administration in WHIM but not WT B cells, independent of whether anti-IgM-mediated BCR crosslinking was present (Figure 2B). These results suggest that the costimulatory signal mediated by CXCR4 may be integrated into the BCR signaling cascade via mTOR.

In both B and T cells, optimal costimulation acts as a checkpoint to avoid antigen activation-induced cell death (AICD). During an immune response, CD40-mediated costimulation of B cells regulates B cell proliferation and differentiation, rescuing B cells from apoptosis (14). To evaluate the effect of WHIM-mutant-CXCR4-mediated costimulation on B cell survival, we analyzed WT and WHIM mouse B cells after anti-IgM activation and costimulation with CXCL12 or anti-CD40. We assessed propensity for apoptosis via Annexin V at 18 h post-stimulation. As expected, anti-CD40-mediated costimulation led to a reduction in Annexin V binding in both WT and WHIM B cells. However, CXCL12, despite the costimulatory effects in WHIM described earlier, was unable to rescue WHIM B cells from anti-IgM-induced pro-apoptotic signals (Figure 2C). Thus, WHIM CXCR4-mediated costimulation leads to a boosting of activation without the pro-survival signals required for optimal B-cell function.

To further assess whether CXCL12 costimulation was insufficient to provide anti-apoptotic signals, we performed assays for Caspase 3/7 activity, as above, in B cells stimulated with anti-IgM in the presence or absence of the chemokine (Figure 2D). While *in vitro* culture reduced the differences in Caspase activity seen immediately after sacrifice (Figure S1D in Supplementary Material) between WT and WHIM, activation by anti-IgM led to significantly higher Caspase activity in WHIM compared to WT. This could not be rescued by CXCL12 costimulation, though it could be rescued by “classical” anti-CD40 costimulation.

Finally, as additional proof, we assessed the mRNA expression of anti-apoptotic gene Bcl-x in activated B cells. *In vitro* culture reduced the difference between WT and WHIM Bcl-x expression observed immediately after sacrifice (Figure S1B in Supplementary Material); yet, as mentioned earlier, CXCL12 and anti-IgM stimulation were unable to rescue the significantly lower levels of expression of the anti-apoptotic gene in WHIM B cells (Figure S2D in Supplementary Material). The different assays described here examine different molecules along the pathway toward apoptosis, acting at different timepoints. However, they all collectively suggest that CXCL12 costimulation in WHIM B cells, despite its boosting of activation levels, cannot convey pro-survival signals.

The ability of WHIM-mutant CXCR4 B cells to be costimulated by CXCL12 was also confirmed in WHIM patients. While BCR triggering induced activation of both WHIM-patient and healthy donor B cells, the administration of CXCL12 in the presence of BCR triggering significantly enhanced activation only in WHIM patient B cells (Figure 2E).

Collectively, these results suggest that WHIM-mutant CXCR4 but not WT-CXCR4 may convey costimulatory signals to B cells. Yet, at least in mice, this is not accompanied by induction of anti-apoptotic signals. Thus WHIM-mutant CXCR4 may be uncoupling costimulatory and survival signals.

### WHIM B Cell Functional Defects Can Be Circumvented by Reducing Antigenic Load during Immunization

**Figure 3:**
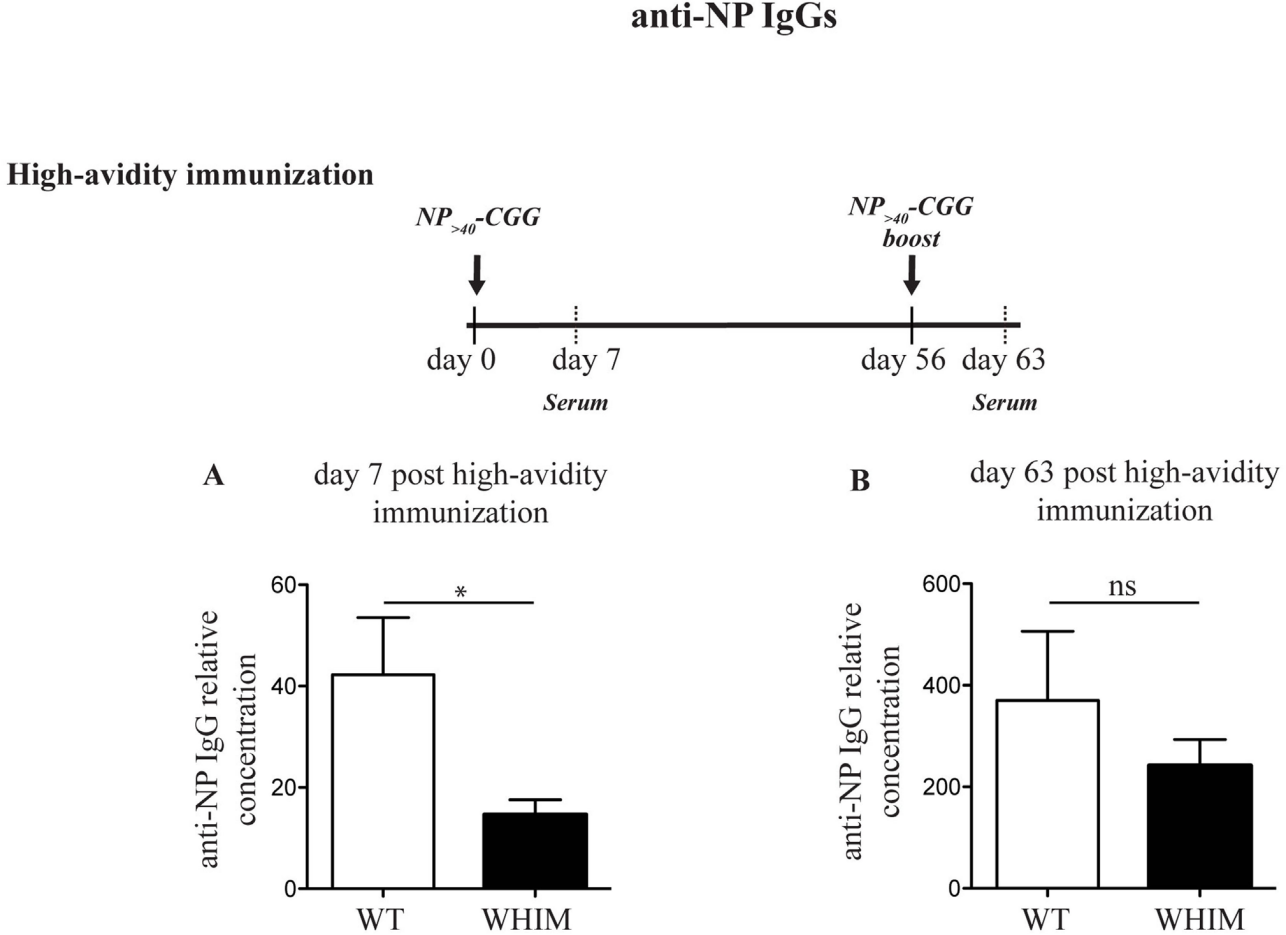

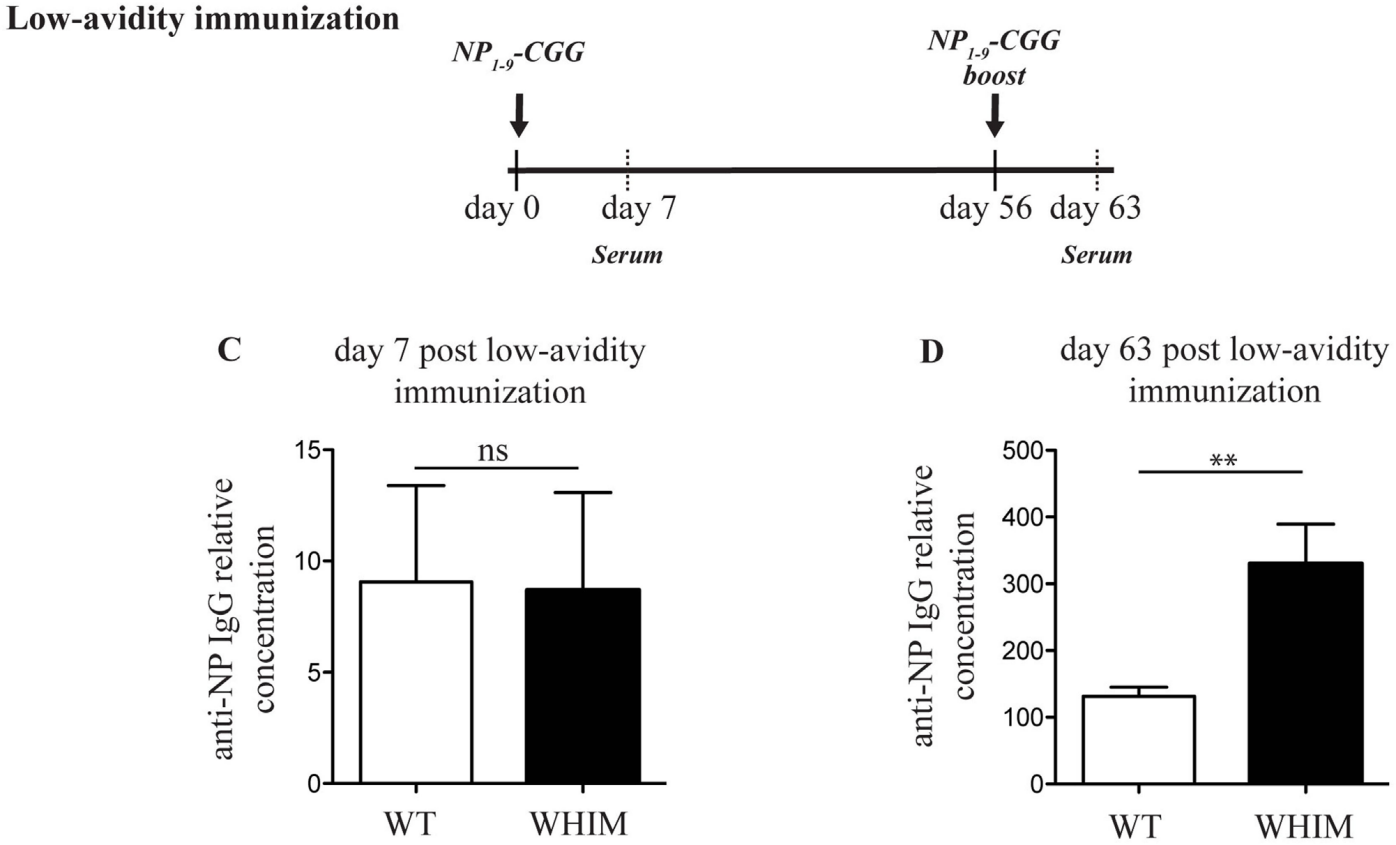
The Warts, Hypogammaglobulinemia, Infections, Myelokathexis (WHIM) B-cell functional defect can be ameliorated by reducing the load of B-cell receptor triggering. Wild-type WT (and) WHIM mice were immunized with high avidity NP (NP_>40_-CGG) **(A,B)** or low-avidity NP (NP_1–9_-CGG) **(C,D)**. The relative concentration of high-affinity antibodies (anti-NP IgG against NP_1–9_–BSA) was evaluated by enzyme-linked immunosorbent assay 7 or 63 days post-primary immunization. Summary results for *n* = 5 WT and 5 WHIM mice (high avidity immunization) and *n* = 6 WT and 6 WHIM mice (low-avidity immunization) are shown. ns: not significant; *P < 0.05; **P < 0.01. Unpaired *t*-test, after assumption of a Gaussian distribution. The anti-NP IgG concentration prior to immunization can be seen in Figure 1H. Details on the immunization protocol and the calculation of relative titers can be found in Section “Experimental Procedures.”

Immunization of WHIM knock-in mice has been recently reported to lead to antigen-specific antibody titers that decrease progressively over time, matching human WHIM patient findings (11). This was despite being characterized by an initial increased accumulation of antigen-specific plasma cells in the spleen though not the bone marrow. The results we report above suggest that aberrant costimulation in WHIM B cells may impair their survival and thus their long-term function. This would be compatible with AICD. A means of circumventing such a hypothesized effect would be to modulate the intensity of the activating signal delivered by antigen, so as to reduce AICD. To test this hypothesis, we immunized WT and WHIM knock-in mice with a carrier protein, chicken gamma globulin (CGG), conjugated to the hapten NP taking advantage of the naturally occurring BCR cross-reactivity to NP (20). We used NP conjugated at two different ratios: higher than 40 NP molecules per CGG (high avidity) and at only 1–9 NP molecules per CGG (low avidity). The former delivers strong BCR triggering, while low-avidity antigen will lead to weak BCR triggering (25). For both immunization protocols, a standard intraperitoneal boost (26) was added at day 56. We examined the production of high-affinity anti-NP IgG antibodies in the serum of immunized animals, 7 days post-immunization and 7 days post-boost (day 63). Day 7 timepoint was chosen as we have previously shown that NP-specific IgG production in mice with antigen-specific (retrogenic) WHIM-mutant T cells is significantly impaired at 7 days post-immunization (12). Indeed, in the WHIM knock-in mice, high avidity NP-CGG immunization led to a reduction in high specificity anti-NP IgG antibodies by day 7 in WHIM knock-in compared to WT mice (Figure 3A). No significant differences were found in high-affinity IgG titers after the boost (Figure 3B). This matches recent findings by Biajoux et al. on WHIM knock-in mice using B-cell chimeras (11). Yet, compatible with our hypothesis, when the low-avidity immunization protocol was applied, even though it produced low overall titers compared to high avidity immunization, differences between WT and WHIM knock-in animals at 7 days post-immunization were eliminated (Figure 3C). Importantly, post-boost (day 63), low-avidity immunization produced significantly higher titers in WHIM-mutant mice than WT animals (Figure 3D). This high-titer antibody response was associated with significantly higher accumulation of class-switched plasma cells in the bone marrow of WHIM-mutant mice, where these cells reside (27) (Figures S3A–D in Supplementary Material), though it is possible that these cells may not be antigen-specific (11). It is noteworthy that our high avidity result differs from the findings by Biajoux et al. on a non-chimeric WHIM knock-in model. However, as our observations on the effects of variations of avidity demonstrate, this may be due to the usage of immunogens featuring different carriers and thus possibly different avidities.

In summary, our findings suggest that the optimal antigenic strength required for the functional and effective activation of WHIM-mutant B cells may be different from WT B cells.

## Discussion

Costimulation of lymphocytes, whether T or B cells, is essential for strengthening the activating signal delivered through the T cell receptor (TCR) or BCR, as well as for activating pro-survival signals, which ensure that a successfully activated T or B cell will avoid AICD. This is mediated via dedicated pairs of receptors, such as CD28–CD80/CD86 and CD40L/CD40 (28). Work from several groups has shown that a subset of chemokines, produced by the APC that is presenting antigen to the T cell, can also mediate T cell costimulation (22, 23, 29–31). However, the hyperfunctional (3, 4, 7) CXCR4 that causes the WHIM syndrome delivers aberrant signals to T cells. It competes with the TCR signals, disrupting T cell-APC synapses, and thus inhibits, instead of enhancing, T cell activation (12). In this report, we identified a novel function of CXCL12 in mediating WHIM B cell costimulation. An important distinction is that for B cells, unlike T cells, antigen activation and (T cell-dependent) costimulation are temporally and spatially distinct events. Unlike T cells that can recognize both antigen and costimulatory receptor ligands on the surface of the same APC, B cells encounter antigen via the BCR independently (32–34) and subsequently proceed into the follicles, where they may encounter a T cell that has been activated by a cognate antigen (35). Only when this opportune encounter has occurred will the B cells receive costimulation. In the case of WHIM, the stability of such B cell–T cell conjugates is known to be impaired (12). On the other hand, a costimulatory effect on B cells mediated by CXCL12, which is abundant in lymphoid organs (36), would have two noteworthy characteristics: first, the interaction between CXCL12 in the microenvironment and WHIM-mutant CXCR4 will not “distract” antigen receptor signaling, as it does in T cells (12), simply because B cell costimulation occurs at a later timepoint and different location from the BCR-triggering event. Second, by being a secreted chemokine, CXCL12 offers a means of costimulation that is not restricted to the cell membrane of a T cell. This would be important in WHIM, as T and B cells are impaired from forming stable synapses (12). Thus, the temporal and spatial characteristics of the CXCL12 signal to WHIM-mutant B cells may enable it to boost rather than inhibit B cell activation *in vivo*, unlike its effects on WHIM-mutant T cells. The above suggest that WHIM-mutant B cells, due to the CXCL12-mediated costimulation, may be spontaneously activated, reminiscent of other “hyperfunctional” immune cell populations observed in WHIM patients (7). The costimulatory effect requires synergy with BCR-triggering signals, as in their absence, CXCL12 alone was insufficient to obtain CD69 up-regulation. However, we also show that, unlike “classical” CD40-CD40L costimulation that both boosts activation and inhibits the induction of apoptosis, CXCL12-mediated costimulation does not convey pro-survival signals, as it did not reduce Annexin V staining in WHIM B cells or did it lead to an increase of anti-apoptotic gene expression. Indeed, we found an increased propensity of *ex vivo* human and mouse WHIM-mutant B cells to undergo apoptosis, even though further survival differences once the cells are grown in culture have not been observed (11). It is noteworthy that increased apoptosis has been previously identified in neutrophil precursor cells of WHIM patients (37). With the important caveat that, due to the rare nature of the WHIM syndrome, our human data are based on a very limited number of patients and thus would have to be confirmed in a larger cohort, we speculate that this “baseline” propensity for apoptosis may contribute to the B cell immunodeficiency that characterizes WHIM patients and mice (10, 11). The full deciphering, at a molecular level, of how CXCL12-mediated costimulation in WHIM uncouples pro-activation from pro-survival signals in B cells, will require further experimentation.

Biajoux et al. recently demonstrated in WHIM knock-in mice that, upon immunization, B cell activation and differentiation were increased, but antigen-specific plasma cell accumulation in the bone marrow was decreased, at the same time as long-term antigen-specific antibody titers were not maintained (11). Our data showing spontaneously higher activation in WHIM patients and WHIM knock-in mice as well as increased low-specificity antibody production in the latter, in the absence of any immunization, are compatible with their findings of increased activation. Yet, they additionally enable the speculation that part of the differences observed post-activation by Biajoux et al. may already be present prior to specific activation. It is likely that the spontaneous B cell responses we observe in the absence of immunization are being mounted against self-antigens. T cell negative selection in the thymus eliminates many self-reactive T cells. The requirement for B cells to be costimulated by cognate-antigen-activated T cells transfers this tolerizing effect of thymic selection to B cells. The B cell costimulation mechanism described here eliminates T cell dependence and thus could possibly open the door to self-reactive B cell responses, offering an explanation for our observations. Coupled with an inability to promote survival, the aberrant B cell costimulation in WHIM may offer a novel explanation for the paradox of increased activation associated with B cell functional defects in WHIM. Further, WHIM-mutant CXCR4 signaling in B cells was found to enhance BCR signaling by further increasing Akt phosphorylation (11). We extended this finding, by observing a CXCL12-dependent phosphorylation of mTOR, which is downstream of Akt. This renders mTOR a putative molecular mediator of the costimulatory effect.

As AICD is dependent on antigenic stimulation load (14), we speculated that CXCL12-mediated costimulation may force WHIM B cells to exceed a threshold of activation, above which survival may be compromised. By reducing the antigenic load with a low-avidity immunization protocol, we were able to enhance post-immunization antibody titers, circumventing a B cell functional defect that is associated with the WHIM syndrome. Yet, an important caveat ought to be mentioned: it remains to be systematically examined whether this finding can be of use for the improvement of immunization strategies for WHIM patients. The only concrete conclusion that can be drawn is that WHIM-mutant B cells may have a different optimal range of antigenic load compared to WT B cells. Indeed, a number of variations between the results of our immunizations and those reported in Ref. (11) may well be due to differences in immunizing antigen avidity, which we have shown here to be a regulating variable.

Nonetheless, the data presented here identify a novel mechanism that may help explain aspects of the multi-faceted adaptive immune response symptoms in the WHIM syndrome. Furthermore, our data demonstrate the existence of novel, unexpected yet interconnected pathways through which chemokines and their receptors, including in B cells (38), may affect the outcome of adaptive immune responses.

## Experimental Procedures

### Patients

Samples from WHIM patients (all with heterozygous CXCR4^C1000T^ mutation) and healthy donors were obtained after informed written consent at Clinica Pediatrica and Humanitas, according to the institutional ethical committee guidelines and the Declaration of Helsinki.

### Animals

Animals were kept in a specific pathogen-free facility and treated according to institutional and national guidelines and regulations, after specific institutional and national authorization, by the Humanitas Clinical and Research Center animal welfare committee and the Ministry of Health, respectively. 8–16-week-old mice were used, age and gender matched within each set of experiments. WHIM-associated mutant CXCR4^+/1013^ Knock-in mice (WHIM knock-in) were generated at the Institut Pasteur as described in Ref. (10) and kindly provided by Dr. F. Arenzana-Seisdedos. Age- and gender-matched WT littermates were used as controls.

### *In Vitro* Activation and *In Vivo* Immunization

For patient and healthy donor *in vitro* B cell activation, peripheral blood mononuclear cells were isolated from fresh blood using Lympholyte gradient (Cedarlane). For mouse *in vitro* B cell activation, single-cell suspensions were obtained by passing cells through 70-µm cell strainers. Red blood cells were lysed in lysis buffer (BD). Highly pure B cells were isolated via magnetic sorting using AutoMACS (Miltenyi Biotec). AffiniPure F(ab′)2 Fragment Goat Anti-Mouse IgM, μ Chain Specific (Jackson ImmunoResearch), AffiniPure F(ab′)2 Fragment Goat Anti-Human IgA + IgD + IgM (H + L) (Jackson ImmunoResearch), mouse anti-CD40 (1 µg/ml; Becton Dickinson), CXCL12 (25 nM; Peprotech), CCL21 (25 nM; Peprotech), AMD3100 (10 µg/ml; Sigma), and anti-CD95 (250 ng/ml, Clone Jo2; BD) were used to stimulate the B cells. For *in vivo* activation, WHIM knock-in or WT mice were immunized by intraperitoneal injection of alum-precipitated 4-hydroxy-3-nitrophenylacetyl (NP)-CGG (NP ratio >40 for high avidity immunization or 1–9 for low-avidity immunization, 100 µg per mouse; Santa Cruz Biotechnology). A boost immunization was performed 56 days after the primary immunization, using intraperitoneally injected NP_>40_-CGG or NP_1–9_-CGG (50 µg per mouse) resuspended in PBS, adapted from Ref. (26).

### Enzyme-Linked Immunosorbent Assay

Enzyme-linked immunosorbent assay for the detection of anti-NP IgG was adapted from Ref. (39). Nunc-Immuno 96-well plates were coated with 10 µg/ml NP–BSA at high avidity (Biosearch, NP:BSA ratio >20) or low avidity (Santa Cruz Biotechnology, NP:BSA ratio 1–9) and detected with anti-mouse IgG-peroxidase (Sigma). NP_>20_–BSA was used to detect high and low affinity antibodies, while NP_1–9_–BSA allows the detection of only high-affinity antibodies (21). Serum from a high avidity NP-CGG-immunized mouse positive control was used to supply a calibration curve for all ELISA and to provide the reference unit for the relative concentration.

### Caspase-Glo 3/7 Assay

The Caspase-Glo 3/7 Assay (Promega) was performed according to manufacturer’s instructions. Briefly, purified B cells were plated in IMDM medium at the concentration of 100,000 cells/100 μl with stimuli [AffiniPure F(ab′)2 Fragment Goat Anti-Mouse IgM, μ Chain Specific (Jackson ImmunoResearch), mouse anti-CD40 (1 µg/ml, Becton Dickinson), and CXCL12 (25 nM, Peprotech)] for 24 h or left untreated. After stimulation, cells were counted and plated at 20,000/well in a white-walled multiwell plate. An equal volume of Caspase-Glo 3/7 Reagent was added to each well for 30 min. Luminescense signal was acquired on a Biotek Synergy 2 luminometer. Background readings were determined from wells containing culture medium without cells.

### Statistical Analysis and Data Handling

Normality testing was applied to discriminate between Gaussian or non-Gaussian distribution of data sets. Gaussian data sets were analyzed via unpaired *t*-test (or parametric one-way or two-way ANOVA for multiple comparisons), while non-Gaussian data sets were analyzed via non-parametric *t*-tests (Mann–Whitney). In all cases, one independent experiment (*n* = 1) refers to one WT and one WHIM animal or patient treated and/or analyzed simultaneously. Independent experiments—due to the non-continuous nature of mouse availability—were processed on different days, introducing experiment-to-experiment variability. For experiments shown in Figures 1B,E,F,I and 2A,E, to enable comparison, values measured on different days were normalized to the WT control of each experiment. In cases where more than one independent experiment was performed on the same day, values were normalized to the mean of WT controls measured on that day (Figures 1B,E,F,I). Statistical analysis was performed using GraphPad Prism. Throughout, *P*-values shown refer to the following: *P < 0.5, **P < 0.01, ***P < 0.001, ns = not significant (*p* > 0.05).

For flow cytometry and real-time qPCR, see Data Sheet S1 in Supplementary Material.

## Ethics Statement

*Patients*: samples from WHIM patients (all with heterozygous CXCR4C1000T mutation) and healthy donors were obtained after informed consent at Clinica Pediatrica and Humanitas, according to the institutional ethical committee guidelines and the Declaration of Helsinki. *Animals*: animals were kept in a specific pathogen-free facility and treated according to institutional and national guidelines and regulations. 8- to 16-week-old mice were used, age and gender matched within each set of experiments. WHIM-associated mutant CXCR4^+^/1013 knock-in mice (WHIM knock-in) were generated at the Institut Pasteur as described in Ref. (10) and kindly provided by Dr. F. Arenzana-Seisdedos. Age- and gender-matched WT littermates were used as controls.

## Author Contributions

GR and EM performed experiments. GR analyzed the data. VL and RB handled the human samples. MK and AV designed experiments. GR, AV, and MK wrote the paper.

## Conflict of Interest Statement

The authors declare that the research was conducted in the absence of any commercial or financial relationships that could be construed as a potential conflict of interest. The reviewer, JB, and handling editor declared their shared affiliation.
