# Supplementary material for: CXCL12 Mediates Aberrant Costimulation of B Lymphocytes in Warts, Hypogammaglobulinemia, Infections, Myelokathexis Immunodeficiency
Source: Front Immunol. 2017 Sep 4;8:1068. doi: 10.3389/fimmu.2017.01068 (PMC5591327; doi:10.3389/fimmu.2017.01068)
Supplement: Supplementary file 1 [file Data_Sheet_1.PDF]

SUPPLEMENTARY INFORMATION

SUPPLEMENTARY EXPERIMENTAL PROCEDURES:

Flow cytometry

Cells were analyzed on a FACSCanto II flow cytometer (BD Biosciences), using Diva Software (BD) and FlowJo. The following antibodies were used: anti-B220 (RA3-6B2, Biolegend), anti-CD138 (281.2, Biolegend), anti-IgM (II/41, eBioscience), anti-CD69 (H1.2F3; BD), anti-CD86 (GL1; BD), anti-human CD19 (eBioscience), anti-human CD69 (FN50, Biolegend), Annexin V (Invitrogen), anti-CD95 (15A7; eBioscience), anti-phospho-mTOR (Ser2448) antibody (Cell Signaling Technology).

For phospho m-TOR analyses, isolated B cells were stimulated with AffiniPure F(ab')$_2$ Fragment Goat Anti- Mouse IgM, μ Chain Specific at 10 μg/mL (Jackson ImmunoResearch) and/or CXCL12 (25nM, Peprotech) for 5 min at 37°C. Cells were then fixed with 1% formalin, permeabilized with ice-cold methanol for 30 minutes and stained with phospho-mTOR (Ser2448) antibody for 30 minutes.

Real-time qPCR

Tissue was homogenized with Tissue lyser in 500μl Trizol with 1 bead/sample. 100μl chloroform was added (chloroform/Trizol 1:6) and samples were incubated for 15 min RT and spun at 10000g for 15min at 4°C. The aqueous phase was collected and RNA extraction was performed using RNeasy micro kit (Qiagen, 74034). cDNA was prepared using High-Capacity cDNA Reverse Transcription Kit (Applied Biosystems). Taqman assays were used (Bcl-x: Mm00437783_m1; Rn18S: Mm03928990_g1; bcl-2: Mm00477631_m1 4331182), with TaqMan Real-Time PCR Master mix, run on an ABI 7900 HT fast real-time PCR system (Applied Biosystems).

SUPPLEMENTARY FIGURE LEGENDS:

Supplementary Figure 1.

A: B cell absolute numbers in the spleens of WT and WHIM knock-in mice. Summary of n=7 WT mice and 6 WHIM mice. ns:not significant, unpaired t-test after positive outcome of normality testing. **B**: Bcl-x mRNA levels in unactivated WT and WHIM knock-in mouse B cells from lymph nodes. Summary of n=4 WT and 4 WHIM mice. **:P<0.01, Unpaired t-test after assumption of gaussian distribution. **C**: Bcl-2 mRNA levels in unactivated WT and WHIM knock-in mouse B cells from spleen. Summary of n=2 WT and 2 WHIM mice. *:P<0.05, Unpaired t-test after assumption of gaussian distribution. **D**: Caspase 3/7 activity in unactivated WT and WHIM knock-in mouse B cells from spleen. Summary of n=3 WT and 3 WHIM mice. *:P<0.05, Unpaired t-test after assumption of gaussian distribution. **E:** Plasma cell frequency in the spleens of unactivated WT and WHIM knock-in mice. Summary of n=7 WT and 7 WHIM mice. ***:P<0.001, Unpaired t-test after positive outcome of normality testing. Percentages were used, rather than absolute numbers, to eliminate variability due to differential cell loss during harvest in independent experiments. **F**: Plasma cell frequency in the bone marrow (BM) of WT and WHIM knock-in mice. Summary of n=7 WT and 7 WHIM mice. ns:not significant, Unpaired T-test after positive outcome of normality testing. Percentages were used rather than absolute numbers, to eliminate variability due to differential cell loss during harvest in independent experiments.

Supplementary Figure 2.

A: Representative experiment out of the 5 experiments shown in Figure 2A. Purified WT (white bars) or WHIM (black bars) splenic B cells were stimulated with anti-IgM (10µg/ml) +/-CXCL12 (25nM) +/-CCL21 (25nM) +/-AMD3100 (10µg/mL) +/- anti-CD40 (1µg/mL). Expression of early activation marker CD69 was evaluated after 18h by FACS. Experimental

triplicates of one representative animal/condition are shown. ns:not significant; ***:P<0.001, (1-way ANOVA and Tukey's post-test).

B: Purified WHIM knock-in or WT splenic B cells were stimulated with anti-IgM (10μg/ml) +/- CXCL12 (25nM) +/- anti-CD40 (1μg/mL) for 18h. CD86 expression was evaluated by FACS. Summary of n=2 WT and 2 WHIM mice. ns:not significant; *:P<0.05, **:P<0.01, (2-way ANOVA and Bonferroni's post-test).

C: Purified WT (white bars) or WHIM (black bars) splenic B cells were stimulated with CXCL12 (25nM) or anti-IgM (10μg/ml) + anti-CD40 (1μg/mL). Expression of early activation marker CD69 was evaluated after 18h by FACS. Summary of n=5 WT and 5 WHIM mice for unactivated and anti-IgM/anti-CD40 group and n=2 WT and 2 WHIM mice for CXCL12. ns:not significant; ***:P<0.001, (2-way ANOVA and Bonferroni's post-test).

D: Bcl-x mRNA levels in WT (white bars) and WHIM (black bars) splenic B cells stimulated with CXCL12 (25nM) and/or anti-IgM (10μg/ml) for 72h. Summary of n=2 WT and 2 WHIM mice. ns:not significant; **:P<0.01, ***:P<0.001, (2-way ANOVA and Bonferroni's post-test).

Supplementary Figure 3.

A: IgM^- (class-switched) $CD138^+$ plasma cell frequency in the bone marrow of WT and WHIM knock-in mice prior to immunization (n=7 WT and 7 WHIM mice), or **B:** 63 days after high avidity NP ($NP_{>40}$ -CGG) immunization (n=5 WT and 5 WHIM mice) or **C**: 63 days after low avidity NP (NP_{1-9} -CGG) immunization (n=6 WT and 6 WHIM mice). ns:not significant; *:P<0.05 Mann-Whitney non-parametric test after negative outcome of normality testing.

D: IgM^- (class-switched) $CD138^+$ plasma cell absolute numbers in the bone marrow of WT and WHIM knock-in mice (n=7 WT and 6 WHIM mice). ns:not significant, Mann-Whitney non-parametric test after negative outcome of normality testing.
