# Supplementary figures and images for: CXCL12 Mediates Aberrant Costimulation of B Lymphocytes in Warts, Hypogammaglobulinemia, Infections, Myelokathexis Immunodeficiency
Source: Front Immunol. 2017 Sep 4;8:1068. doi: 10.3389/fimmu.2017.01068 (PMC5591327; doi:10.3389/fimmu.2017.01068)

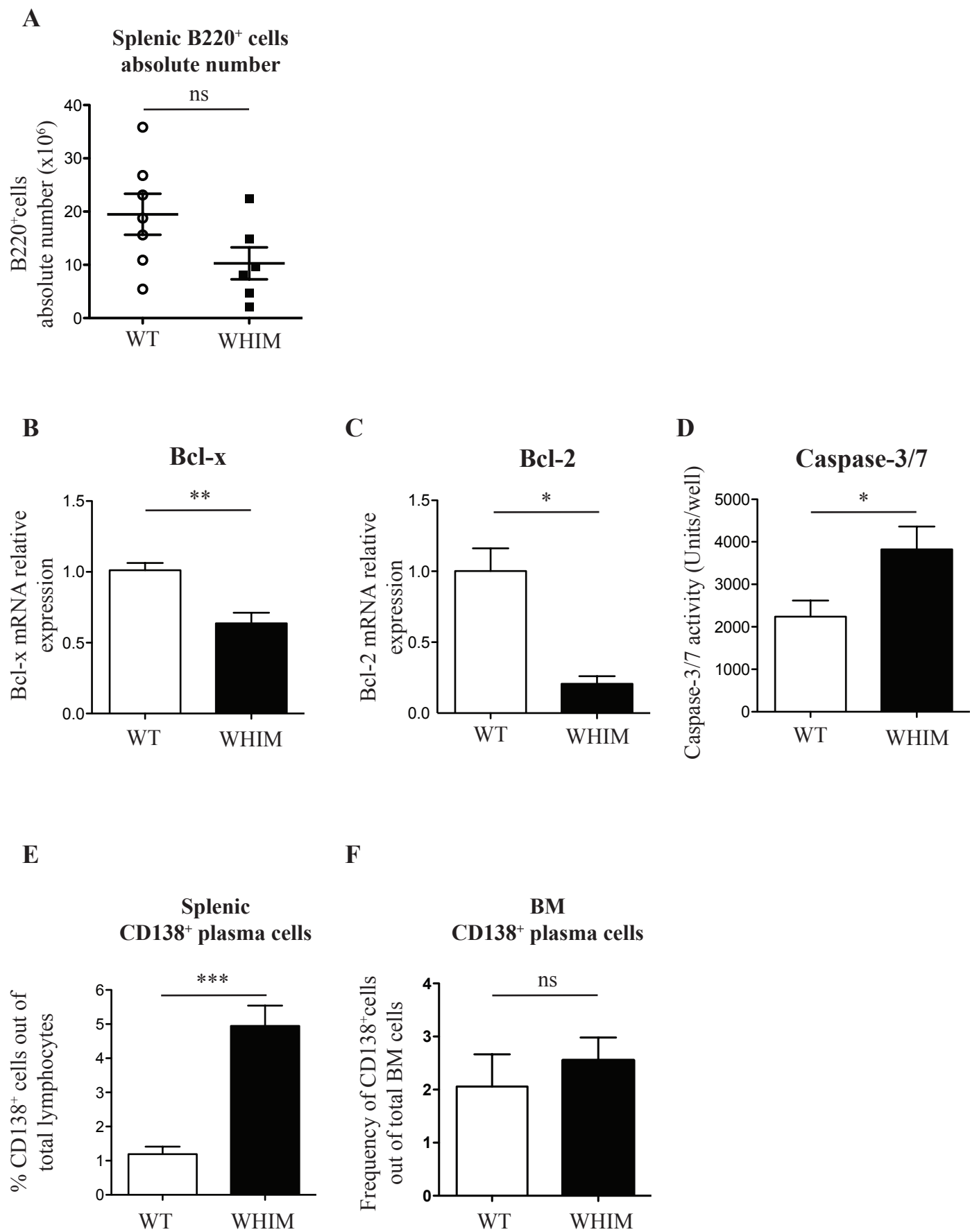

**Supplementary Figure 1**

Supplement: Supplementary file 2 [file Image_1.PDF]

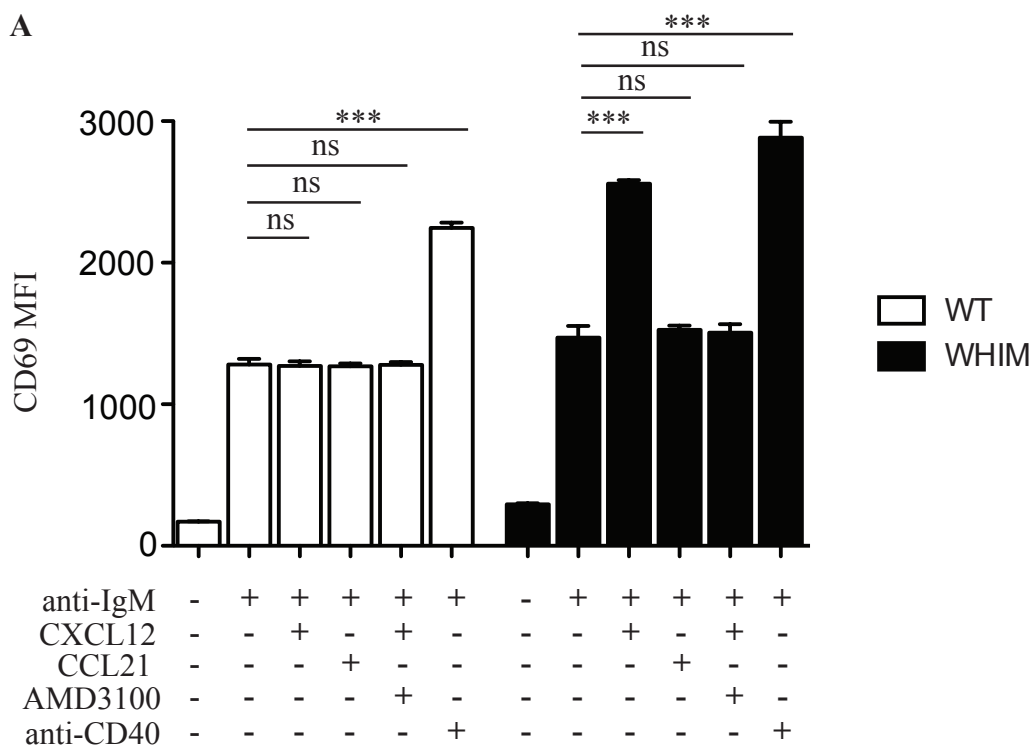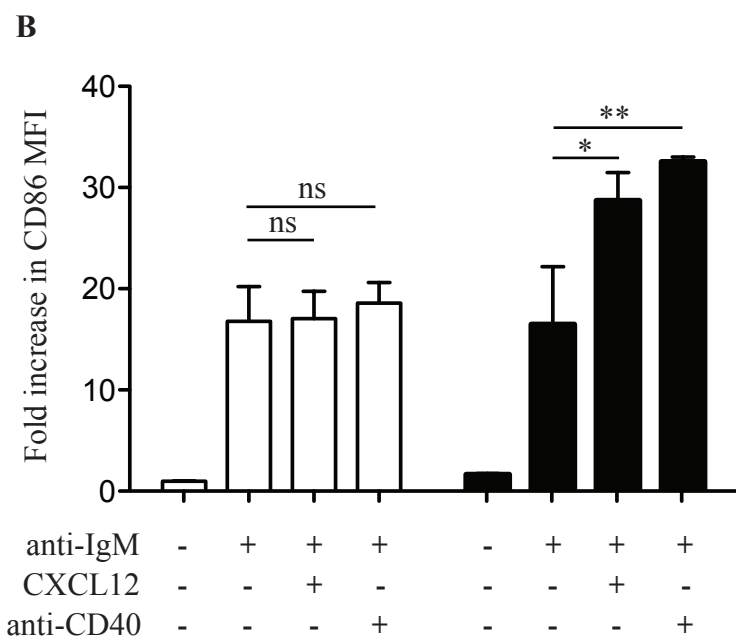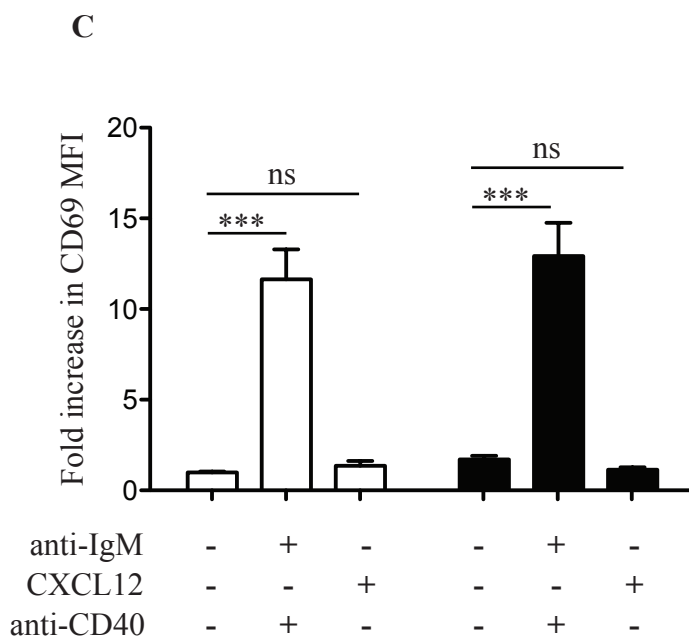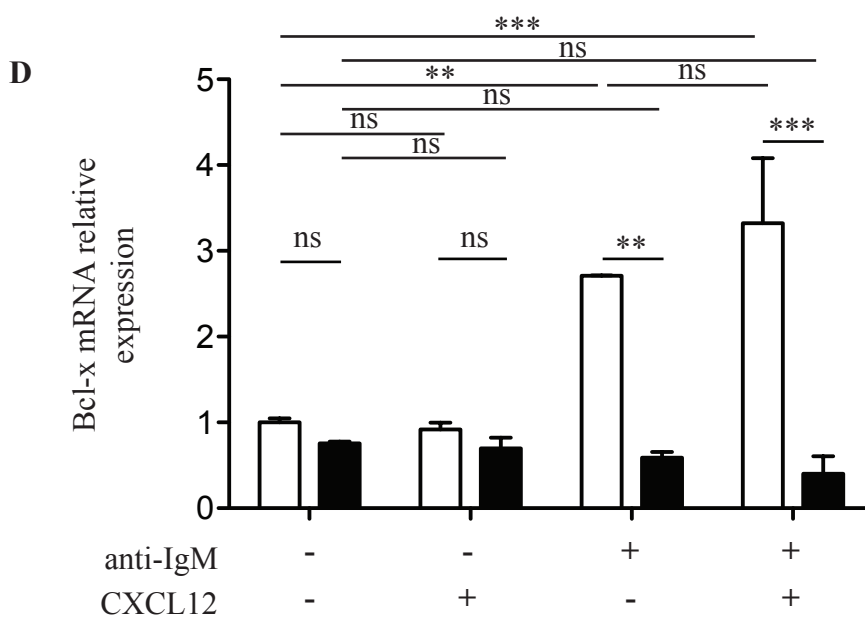

Supplementary Figure 2

Supplement: Supplementary file 3 [file Image_2.PDF]

Switched plasma cells in the bone marrow

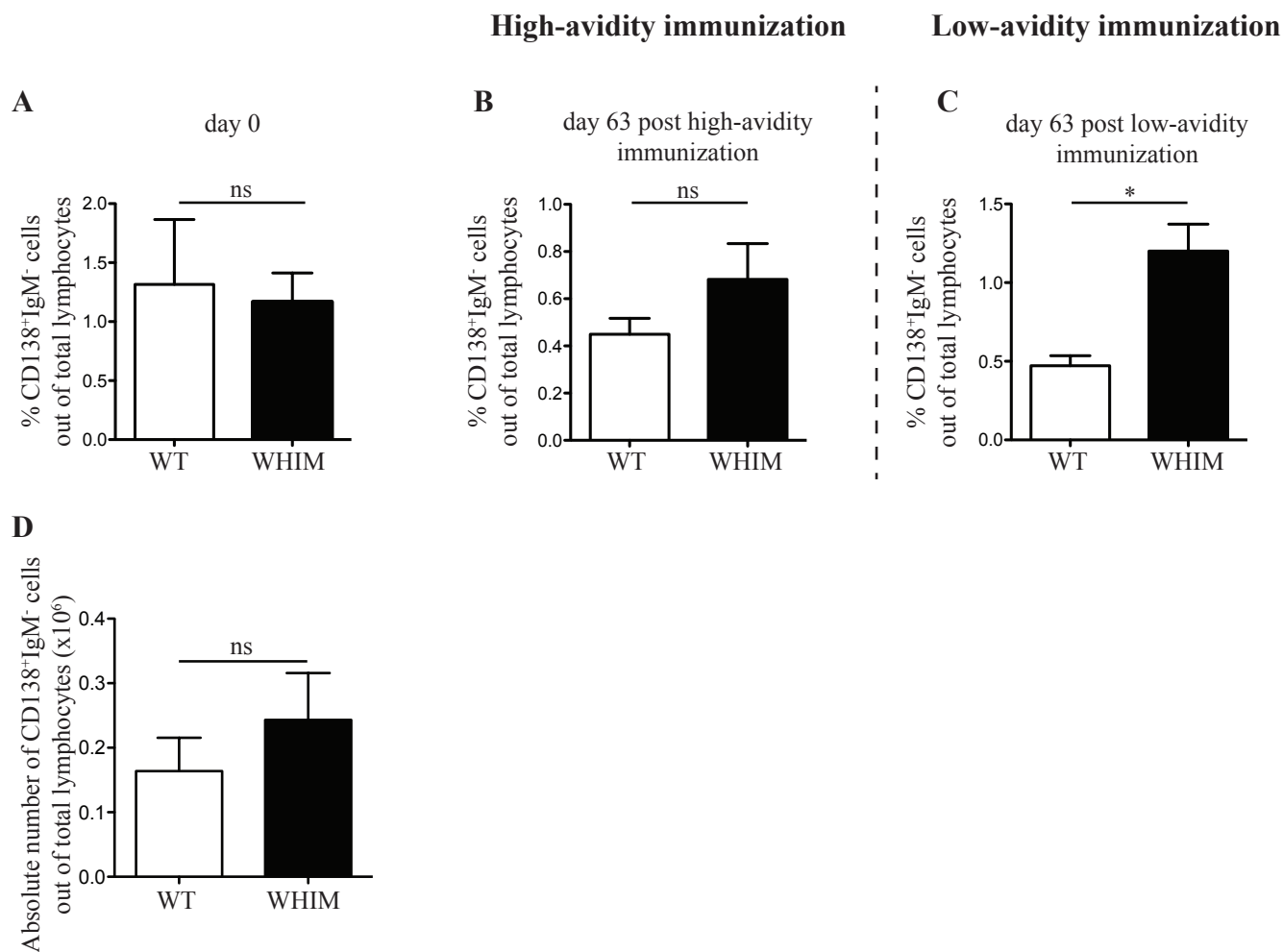

Supplementary Figure 3

Supplement: Supplementary file 4 [file Image_3.PDF]
